# Chicago Medical Society

**Published:** 1889-03

**Authors:** 


*Regular Meeting, January 8, 1889.*

The President in the Chair.

Dr. F. S. Johnson read a paper entitled

PATHOLOGY OF DIPHTHERIA.

Diphtheria is an acute specific infectious and contagious disease, with local and general manifestations and characteristics. The local lesions are found in the upper air passages, especially in the pharynx—but also in nose, larynx, trachea and deeper parts of the respiratory tract. These may, however, occur primarily in other parts of the body. The general manifestations are fever and concomitant symptoms. The local manifestations are inflammation and the formation of a false membrane on the surface, or in the superficial portions of the tissue.

From the clinical standpoint, it is difficult to say whether diphtheria is primarily a general or local disease. The general symptoms usually appear before the local trouble is manifested. On the other hand, the local trouble sometimes exists with scarcely noticeable general symptoms. The poison seems to usually gain access to the body with the inspired air.

By analogy, the pathogenesis of the disease is at the present time commonly attributed to micro-organisms, although no form has yet been found to satisfy the conditions formulated by Koch, and held by all experimenters as necessary for proof, viz: Micro-organisms are sometimes found in the kidneys, but according to Birch-Hirschfeld only in cases of severe sepsis.

*First.*—The micro-organism must be present in all cases of the disease.

*Second.*—Pure cultures of this micro-organism must be capable of producing the disease.

*Third.*—The micro-organism must be found in the tissue of the animal in which the disease is experimentally produced.

No one form has yet been found to be invariably present. No form has been found which always produces the characteristic phenomena of the disease and which does not give rise to other forms of poisoning. In experiments upon animals the results of inoculation have, in most instances, either been negative or more nearly resembling the phenomena of pyæmia and septicæmia. All the forms found in diphtheritic membranes have been found in the human mouth under normal conditions. The recent researches into the mode of action of micro-organisms in disease have led to a change in the generally accepted views upon the subject. It is now held that micro-organisms do not in themselves act as poisons, or to any extent mechanically by forming capillary emboli, but that by the splitting up of proteid substances they produce ptomaines and set free ferments, and that these are agents in the causation of disease.

Anatomically classified, the local phenomena of diphtheria are:

Catarrhal inflammation.

Croupous inflammation.

Diphtheritic inflammation.

Topographically classified, the disease is diphtheria when it occurs in the pharynx and nose; croupous or laryngeal diphtheria when it occurs in the larynx.

It is proper here to mention other specific disease forms with similar or identical local manifestations:

Diphtheritic inflammations, occurring in the course of the exanthematous fevers.

Wound diphtheria.

Dysentery.

Puerperal Endometritis.

Diphtheritic Cystitis.

Etiologically considered, there may be as many forms as there are agents capable of destroying tissue and exciting inflammation.

In extremely mild cases the local changes are indistinguishable from those of simple catarrhal inflammation, and anatomically they belong to this class. The damage to the mucous membrane by the specific poison is here not sufficient to destroy any part of it, but simply to excite a moderate inflammation. And under these conditions the exudations from the blood-vessels in reaching the surface is filtered through the epithelium of the mucous membrane and is thereby rendered non-coagulable, and is removed from the parts affected in the same manner as are the normal secretions. In these cases there is no false membrane, though the cause is the specific poison of diphtheria. To what extent these mild cases occur it is impossible to say, because the presence or absence of a false membrane usually determines the diagnosis.

The false membrane or diphtheritic membrane is the result of combined inflammation, tissue death and coagulation within the densely infiltrated tissue. The thickness of the membrane varies with the depth to which the tissue is destroyed. The essential anatomical distinction between simple catarrh and diphtheritic forms of inflammation of mucous membranes lies in death of the tissue, especially of the epithelium.

When the epithelium has been removed in whatever way, inflammatory exudations upon the denuded surface will coagulate, thus forming a false membrane. The destruction of the epithelium by chemical agents and heat is followed by the formation of a coagulum upon the surface; or in other words, a false membrane.

Anatomically, there are two varieties of pseudo-membranes—croupous and diphtheritic. In the former the epithelium only is destroyed, and the depth of the pseudo-membrane is limited by the basement membrane of the mucosa. In the latter the underlying tissues also suffer death. Mucosa, sub-mucosa, and even the deeper tissues, are involved in the formation of the pseudo-membrane.

In the pathological processes, the etiological agent, viz: the diphtheritic poison, is the prime factor. It seriously impairs the vitality of the tissues or destroys them outright. This injury produces inflammation, and the already damaged tissues are infiltrated and swollen by the copious exudation from the blood-vessels. Death of the entire mass soon follows, and with it coagulation. This coagulation of tissue and inflammatory exudate, constitutes the false membrane.

The greater the injury to the tissue, the deeper the processes reach and the more intense the inflammation. The character of the exudation depends upon this condition. In the milder forms the exudate is made up almost wholly of leucocytes and plasma; in the severer forms large numbers of red blood-corpuscles are extravasated, and the infiltration is more or less hæmorrhagic in character.

The diphtheritic membrane usually appears first upon the tonsils, sometimes upon the soft palate, or even in the nose, and not infrequently in the larynx. At first it is a thin, irregularly oval white or grayish patch, or even merely a lace-like layer of fibrinous shreds upon the surface. When fairly formed the membrane is tough and firmly adherent.

In croupous membranes the firmness of the attachments depends in a measure upon the character of the underlying mucous membrane. When this is closely adherent to the parts beneath, the pseudo-membrane is less easily cast off. Thus, in the larynx, pseudo-membranes are more firmly attached to the vocal chords and to the lower surface of the epiglottis than over other parts. As the local changes go on, the tissue becomes involved to a greater depth, and this extreme infiltration adds to the bulk of coagulable material. Thus, what began as a croupous membrane may become diphtheritic in character. As it undergoes degeneration it becomes yellowish and fatty. It is gradually cast off as a whole, or in shreds or layers.

In severer cases the coagulum contains a variable proportion of red blood-corpuscles, and the membrane is darker and greenish.

In the most severe forms the affection spreads rapidly from the beginning; the membrane is dirty, hæmorrhagic in appearance, and foul smelling.

Gangrene seldom occurs, and when present it is probably not due to the intensity of inflammatory processes, but to the direct effect of the virulent poison.

The false-membranes may sometimes, in all probability, be the seat of invasion by micro-organism capable of causing pyæmia or septicæmia. In these cases the individuals may suffer from this infection in addition to the existing diphtheria, in the same manner as though infected through any exposed wound. This condition may seriously complicate the symptoms of the primary disease. Gangrene, too, may be due to other causes than the diphtheritic poison acting upon the already damaged tissues.

Histologically, pseudo-membranes consist of homogeneous flakes and strands and fibrinous threads, enmeshing leucocytes and red blood-corpuscles. Frequently the quantity of leucocytes is so great as to obscure the fibrinous coagulum. Remnants of the former tissues are also included in the membrane. As the membrane undergoes degeneration the fibrin becomes finely granular, and the appearance of a net-work is lost. The tissues immediately underlying the false membrane are densely infiltrated. On tearing off the latter, the surface is mottled red and pale; there are seen bleeding points, interstitial hæmorrhages and shreds, and masses of adherent and more or less degenerated pseudo-membrane.

The membrane may extend from the primary seat to surrounding parts, as from the tonsils to other parts of the pharynx into the eustachian tubes and the nose, seldom into the œsophagus; more frequently into the larynx, trachea and bronchi. When stenosis of the larynx occurs, there is varying acute emphysema of the upper lobes, and hyperæmia of the lower lobes of the lungs. If, when in this condition, the smaller bronchi are invaded or are closed by aspiration of bits of pseudo-membrane, there is occlusion of the bronchi and bronchioles, and consequent atelectasia and lobular pneumonia. These conditions rarely occur in the upper lobes, because of the emphysema and consequent anæmia. Under these circumstances, if air is freely admitted to the lungs by tracheotomy, the upper lobes are more readily infected.

With general infection of the body are associated more or less extensive pathological changes in the various tissues and organs. Some of these are manifested chiefly by derangement of physiological functions.

The anatomical changes result from the direct affect of the noxa upon the tissues. The most constantly observed, secondary local infection in pharyngeal diphtheria is that of the lymphatics behind the jaw, and the superficial glands of the neck. These are often highly inflamed and much swollen.

In laryngeal diphtheria the swelling of the superficial glands is usually absent, since the lymphatics of the larynx are more deeply situated, and swelling of them often escapes notice.

Hæmorrhages are common in the various organs, and from serous surfaces. The heart muscle and voluntary muscles undergo fatty changes. Waxy degeneration has also been observed.

In the kidneys, even in the lighter cases, there is cloudy swelling of the epithelium. In severe cases there is frequently glomento-nephritis, hæmorrhage, extensive degeneration of the epithelium in the convoluted tubes, and sometimes diffuse nephritis. The spleen is hyperæmic, the malpighian bodies often enlarged, as are the lymphatics of the body generally. In the mucous membrane of the alimentary canal the lymph follicles are often swollen; sometimes there is superficial ulceration. In the liver there is a more or less degree of fatty degeneration. Diphtheritic inflammation of the stomach seldom occurs, and then confined to the cardiac portion. In the brain and cord meningeal and parenchymatous hæmorrhages sometimes occur.

Klebs has found accumulations of micro-organisms in the perivascular lymph spaces of the brain, and Dejerine has found accumulations of white and red blood-corpuscles similarly situated in the spinal cord.

In three cases of extensive diphtheritic paralysis he found advanced degeneration of the nerve fibres of the anterior root, and degeneration of the ganglion cells in the anterior horns.

In most of the cases of paralysis the disturbance is referable to degenerative changes in the peripheral nerves.

Dr. D. R. Brower read a paper entitled

NERVOUS SEQUELÆ OF DIPHTHERIA.

The morbid phenomena left by diphtheria in the nervous system are paralysis of motion and sensation, chorea, epilepsy, and insanity. These sequelæ occur more frequently in this disease than in all the other acute diseases combined, and they are always in the way of a favorable prognosis, for at least six weeks, no matter how mild the primary disease may have been.

Paralysis occurs in about forty per cent. of all the cases treated. It may commence as early as the second day of the diphtheria, but usually does not appear until the second or third week after the termination of the throat symptoms. This paralysis bears no direct relation, as to time of development or intensity, to the severity of the primary disease, or the previous health of the individual. A very mild case may be followed by the severest paralysis. It is much more frequent in adults; indeed, the older the patient the greater the danger; although it may occur at any time from two years upward.

The paralysis usually begins in the velum palati, the place of the primary morbid activities, being the place of the beginning of the nervous sequelæ. This produces a nasal tone in the voice, and a partial regurgitation of liquids through the nose during deglutition, and along with this motor impairment there is ordinarily anæsthesia. Paralysis of the pharynx is not so frequent as paralysis of the palate, but more dangerous, and it may be so severe as to make swallowing impossible. The superior laryngeal nerve may be paralyzed, producing anæsthesia of the mucous membrane of the larynx, and destroying, in part, the functions of the epiglottis; so that food is very liable to enter the larynx and trachea. If the particles are small they may reach the bronchi, causing pneumonia; if large, they may occlude the trachea and produce sudden death. The paralysis may also include the vocal cords, making phonation impossible, and so far interfering with respiration as to make intubation or tracheotomy necessary.

Paralysis of the accommodation of the eye, due to paralysis of the ciliary muscle, may occur, and will be overcome by the use of convex glasses. The motor-occuli nerve may also be paralyzed, producing diplopia, strabismus and ptosis.

Paralysis of the lower limbs may succeed that of the soft palate. The patient will first complain of numbness, formication, tingling and pain, and then, very soon, muscular weakness will be manifested. The loss of power is rarely complete, the condition being more a paresis than a paralysis, and with it there is usually ataxia. The case may at this time easily be mistaken for locomotor ataxia, especially when, as is usual, you find absence of patella tendon reflex. This ataxia will continue often for weeks after all paresis has disappeared. If the paralysis continues to advance, the upper extremities may next be involved, and here, as in the lower extremities, disorders of sensation usually precede those of motion—and ataxia is usually present here also.

The muscles of the trunk and neck, and the sphincters of rectum and bladder, may, later along, be paralyzed, although very rarely.

The diaphragm and heart are sometimes involved; the former, while grave, is not so serious as the latter. Heart failure is a cause of death in many of the malignant cases in the primary disease, but sudden death not infrequently occurs from this cause, after convalescence has been well established. This fatal accident is sometimes preceded by præcordeal distress, dyspnœa, slowness and irregularity of pulse, and badly accentuated heart sounds.

The earliest, most frequent and most persistent phenomenon of perverted nervous action, is the loss of patella tendon reflex. This may occur very early in the primary disease, but usually does not occur until the second week, and may continue several weeks after otherwise complete recovery.

The pathological anatomy of post-diphtheritic paralysis is yet uncertain. In some cases the lesion is without doubt a peripheral parenchymatous neuritis, in other cases the paralysis depends upon a poliomyelitis anterior; and more rarely cerebral hæmorrhage or meningitis is found as the pathological basis of the symptoms. These several lesions can not be present in the majority of cases, for the paralysis is often too transitory in its duration and too variable in its location, to be due to any fixed structural change in the tissue of the nervous system. The micro-organisms that constitute the materies morbi of the primary disease are the causes of this phenomena. Either directly by their presence in the nerve elements of the central or peripheral systems—or in the blood-vessels that supply them, or else they produce some poisonous products the more or less sudden development of which destroys or disturbs the functions of the nervous system.

The prognosis of the paralysis is favorable except it involves the muscles of deglutition, respiration or the heart. The sooner the paralysis occurs, the more unfavorable the prognosis. The duration of the sequalæ is very variable.

The treatment of post-diphtheria paralysis must be tonic and mildly alterative, an abundance of easily digested nourishment, a moderate amount of alcoholic stimulants, and when there is paralysis of pharynx, epiglottis or upper part of the larnyx, the early use of an œsophageal tube is demanded, as well as feeding by the rectum.

Strychnia should be used with some caution in the beginning of the paralysis lest by producing an undue determination of blood to the spinal cord we increase the pathological conditions. Iron and quinine will usually be of service from the beginning.

Mild alteratives, the iodides and mercury are indicated in the majority of cases, along with the tonics, to stimulate the absorbents, and thereby hasten the removal of morbid products from the nervous system.

Electricity in the form of the *mildest* current that will produce muscular contraction is of service. In some cases the faradic current is sufficient, but more frequently the interrupted galvanic current is necessary for this purpose.

Massage will be of service if the paralyzed muscles are accessible to the manipulations.

Dr. A. H. Foster read a paper entitled

THERAPEUTICS OF DIPHTHERIA.

Clinically we meet two varieties, the fibrous and the putrid. Generally the fibrous has no odor, or, if any, only a faint sour-milk smell, until fatal exhaustion and sepsis have supervened. When this occurs in the fauces or larynx, and is seen early, mercury, in frequent liberal doses, is my main reliance. The system in diphtheria, as in syphilis, seems very tolerant of mercury until an impression is made; then dosage by it must be guarded. While giving the mercury add nourishment and stimulants, such as whisky, and quinine by injection, if not borne by the stomach.

Bind turpentine and camphorated oil, equal parts, freely about the neck. Keep a large dish of hot water steaming beside the bed, with turpentine constantly floating and vaporizing upon it, using eucalyptus oil with it if desired. Keep lime water with benzoate of soda in solution, by means of an atomizer, steaming into the patient's face constantly, keeping the room of uniformly rather high temperature.

Very little confidence is placed in topical applications for the fibrous forms. Papoid ℈i to ℥i glycerine is brushed on every hour; a brush moistened in glycerine and rolled in sulphur is applied often, or sulphurous acid and glycerine—equal parts.

Three cases this season of laryngeal form, in imminent peril, and the surgeon in waiting, have recovered under the above treatment with the aid of bountiful additional warm vapor in the room.

Of course perfect quiet in bed for days or weeks following the disappearance of the exudation is enjoined, with a judicious administration of bitter and ferrugineous tonics.

In the putrid form, with or without nasal complications—in patients not too young—a $\frac{1}{4000}$ to $\frac{1}{8000}$ solution of the bichloride of mercury is injected through the nose into the throat every six to twelve hours, to cleanse that part of the fauces not reached by the mouth.

By way of the mouth the fauces are mopped with a stronger solution of the bichloride at the same time. The tincture of iron and chlorate of potass. solution is used as liberally as the stomach will allow. The disinfecting turpentine vapor is kept constantly forming, often from a large basin resting in a notched piece of stovepipe, over a kerosene lamp. The limewater spray is used as freely as possible. The turpentine and oil is kept upon the neck, and with flaxseed poultices, if the glands are much involved.

The following is from Dr. J. W. Tope, of Oak Park, Ill.:

"*Dear Dr. Foster:* Yours of to day just at hand. In reply I will state briefly my line of treatment. If I see the case early —within the first twenty-four hours—I generally put the patient on large doses of sodium benz., giving it every hour or two for twenty-four, sometimes forty-eight hours. At the first appearance of deposit either on tonsils or elsewhere, I order a sat. solution of acid. borici to be used as a gargle, also to be thrown into each nostril (warm) with syringe every hour or two, according to the urgency of the case. I also apply to every diphtheritic patch in reach the bichloride solution, one to one hundred in alcohol every hour or two, taking care to squeeze out of the cotton (I use absorbent cotton) all superfluous solution so that the patient may not swallow it. I use the boracic acid solution in all cases, because I think that what appears to be diphtheritic deposit only on fauces or tonsils, in many cases, does extend behind the soft palate. Generally after using the sodium benz. for a day or two, I put the patient on large doses of iron. Generally now use whisky liberally—as soon as the least indication of the larynx becoming involved I begin the use of mercury. Generally use the bichloride. When giving benz. sod. use from ten to thirty grains, according to age. In putrid cases, if patient is old enough to keep the injection from being swallowed, I use an injection through the nostrils of one to 4,000 of bichloride of mercury every eight hours. I do not use turpentine, but eucalypt. in vapor. When I use iron I have it given largely diluted, and with a wooden spoon. Use the one to 100 bichloride in alcohol only with swab."

Dr. E. Fletcher Ingals: I have been asked to speak upon the subject of intubation as compared with tracheotomy for the relief of diphtheritic laryngitis. I have recently found, in looking over the literature, that O'Dwyer's method has steadily gained ground with the profession in this country during the last year. Numerous papers have appeared, showing that many physicians are adopting this method, and I think we may say, without hesitation, that it has reached the point where it may be termed a recognized procedure. In Europe the status of intubation is now about the same that it was in this country two years ago, but the reason for this does not seem to be found either in the operation or in its results.

Many experiments have been made with a view to improving this operation, but so far as I can learn the majority of operators have concluded that O'Dwyer's methods and instruments have not been improved. There appeared, during the past year, a short but interesting article, by Dr. James Rich, of London, on "Naso-Pharyngeal Intubation," in which he recommends the passing of a catheter through the nostril and nares into the larynx, and retaining it in that position so long as necessary for respiration. He recommends, at the same time, the passing of a small œsophageal tube through the other nostril into the stomach, for the purpose of feeding the patient. He had tried the method on four cases. It worked very satisfactorily for a few days, but unfortunately none of his cases recovered. This is not new, but is of interest as showing the efforts being made to improve the operation.

The operation of intubation, as we understand it, or O'Dwyer's method, seems to be especially adapted to those cases which can not be well cared for by nurses or physicians. This is exactly contrary to the suggestion of some foreign physicians, who urge as one of the greatest objections to this measure that the physician or nurse must be constantly in attendance, capable of introducing the tube. The experience of physicians in this country indicates that the laryngeal tube does not require as much attention as the tracheotomy tube, and that when expelled by cough there is nearly always ample time to summon the physician to reintroduce it.

Some one has specially recommended intubation in cases which occur with epidemics of measles, as in these cases tracheotomy is seldom or never successful. It is in these cases, particularly, that considerable ulceration of the larynx, or the trachea, has been set up by the tube and intubation has not proved very efficacious.

I think it is well established that intubation is to be preferred to tracheotomy in very young children. It has also been recommended for adults, whom it has been stated never recover when tracheotomized for diphtheria. I can not vouch for the correctness of this statement. One of the most prominent dangers of this operation is that of crowding membrane down before the tube, and thus preventing respiration. However, this is not a very frequent accident. O'Dwyer says that out of 200 cases he has only had the accident occur twice, and in these two cases, on withdrawal of the tube, the membrane was immediately coughed up. This has been the experience of some others. But in some cases the membrane will not be coughed out. In such a case I believe we should do tracheotomy immediately.

In this I am not in accord with my friend Dr. Waxham, who recommends trying to remove the false membrane with forceps. The length of time the tube may remain in the larynx should be considered when comparing this operation with tracheotomy, as it has been thought by some that the laryngeal tube could not be left long enough to accomplish the results desired. There is no difficulty from this source, as it has been found that the tube may be left as long as necessary, without causing complications, excepting those cases complicating measles, in which limited ulceration is liable to occur. The tube will generally remain from three to five or six days, but it is often coughed out the third or fourth day, and in many instances it is not necessary to return it; but in others it must be reintroduced and worn until the fifth or sixth day before it can be finally removed, and a few will have to wear it even longer.

It has been urged against intubation that particles of food find their way into the trachea and then into the lungs, causing pneumonia by aspiration, or "*Schluck-pneumonie.*" I think it a common belief with the profession that this is the usual cause of pneumonia after intubation. I have held this view myself, and still hold it with reference to some cases, but it is a singular thing that in 116 autopsies recorded by Dr. Northrup, of New York, not a particle of food could be found in any portion of the respiratory tract. This would seem to prove the danger of pneumonia by aspiration has been much overestimated. The risk of tubes slipping into the trachea has been urged against intubation, and I think it is a danger that has been much underestimated by the profession. Several instances in which this accident has happened have been published; I know of some cases that have not been reported, and have no doubt that others have occurred. This danger can only be avoided by using a tube of proper size. Those who are in favor of tubes with small heads do not consider this danger of much importance, but I would urge upon you the necessity of using tubes of proper size with the large head recommended by O'Dwyer. It was urged early in the history of intubation that the tubes were liable to become stopped up, and might cause suffocation before they could be removed. This accident is rare but it may occur slowly by drying of the secretions or suddenly by loosened membrane. One case is reported in which the child vomited and portions of the contents of the stomach were drawn into the tube so that the patient would have suffocated if the physician had not been present. If the tube becomes occluded slowly the gradually increasing dyspnœa will attract the nurse's attention so that the physician may be called to remove it, but if it becomes stopped quickly the only chance for the patient is in coughing it out, therefore a tube which does not fit tightly should be used. Until recently we all believed that a child three or four years of age could not breathe through a tube less than one-fourth of an inch in diameter, but we have found that this impression was erroneous, and that they may get sufficient air through an opening not more than one-third of the size. It is claimed by O'Dwyer that the small openings are better than the large ones, because patients wearing such tubes can cough more forcibly, and expectorate more readily.

A tube which will slip into the larynx without much force should be used in preference to one which has to be crowded into the glottis. It is especially important that the physician should remember that the tube merely allows the entrance of air, and has nothing whatever to do in curing the patient. I believe that in many instances the physician gives up his work as soon as the operation has been done, and does not continue proper medication. During the past year the discovery of the new method of feeding, which was presented to this society by Dr. Casselberry (and which I think he says was suggested by Dr. Cary), has done very much to remove from intubation one of its most prominent objections. Before this method of administering fluid was discovered it was necessary either to feed the child solids or soft solids, or to nourish it by enemas, and to withhold fluids. By this method we find that the child placed upon its back, with the head low, can swallow fluids readily without the danger of its running into the trachea and causing cough and possibly pneumonia. I believe that with the adoption of this method of feeding, the operation will soon become much more general than it now is, and as it becomes more general I think we will find that the results are better than they have been.

In the spring of 1887, I gathered together the reports of 514 cases of intubation, in which the percentage of recoveries was 26.07 per cent. Dr. Waxham gathered together for the International Medical Congress, in the fall of the same year, the records of 1,072 cases, with a percentage of recoveries of 26.77, showing in that short space of time a considerable improvement in the percentage of recoveries. Dr. O'Dwyer reported a series of 100 cases, with a percentage of 27 recoveries. And the latest written report from Dr. Waxham is 169 cases with 29 percentage of recoveries.

The percentage from tracheotomy varies according to different reports. Where large numbers have been collected it is from 26.25 to 28, thus showing that the percentage of recoveries from the two operations is essentially the same. There are instances in which the percentage of recoveries from tracheotomy has been much less, as there are those in which the percentage of recoveries from intubation has been equally unfavorable, but there are others in which results have been much better. Some operators have had nearly 50 per cent. of their cases recover after tracheotomy; but the most comprehensive statistics place the recoveries from this operation fairly, at from 26.25 to 28 per cent., and the largest collection of cases of intubation place its successes at 26.77 per cent. I do not believe intubation adapted for all cases, but I do believe it should be used in preference to tracheotomy in nearly every case in the beginning. If it does not relieve the dyspnœa, tracheotomy should be resorted to. Of course intubation should not be used where the obstruction to respiration is in the fauces instead of the larynx, as occasionally happens.

Dr. F. E. Waxham: In looking over the records of my cases to-day I find I have performed intubation 180 times, with 56 recoveries, or something over 31 per cent. My *first* 150 cases were reported before the American Medical Association, and have been fully published. Since then, within the last six months, I have had 30 cases, with 15 recoveries, or 50 per cent., and I believe that the skill and the judgment that comes with increased experience will enable us to continue to save at least 50 per cent. of our cases. In looking over the records I find that many cases have been saved only by the most judicious and watchful care, and, again, I find that a few cases have been lost where, if better judgment had been exercised, I am sure the patients might have recovered. For example, not long since I performed the operation on a little boy 12 years old. It was so perfectly evident that there was membrane below the tube that a thread was left attached to it in order that the parents might remove the tube at a moment's notice, in case there was detachment below the tube. All went well for two days, when the child accidentally severed the string by chewing upon it, and, about two hours later, detachment of membrane occurred, and the child died in a few moments. In this case, if the tube had been removed at the moment or soon after the thread was severed, undoubtedly the child would have recovered. The membrane would have been expelled, or, if not, a smaller tube should have been introduced, and this would have been expelled when the membrane became detached below it, or, as Dr. Ingals suggests, tracheotomy should have been performed. Dr. Jacobi, of New York, than whom we have no better authority, after having performed tracheotomy 500 times, now recommends intubation. Such is the conclusion of a man who, perhaps, has performed tracheotomy more times than any one else in America. In the last issue of the *Journal of Pediatrics*, Dr. Huber, of New York, reports 94 cases, with 37 recoveries, or 44 per cent.; so it seems that the record of intubation is improving.

In regard to feeding the patients, a great deal of judgment and discretion should be used in every case. We must find the idiosyncracies of each patient. Many patients will not swallow in the inclined position; we cannot induce them, we cannot compel them to swallow while standing on their heads; and when this is true we must remove the tube and employ one with an artificial epiglottis, or else continue to use semi-solid food. It should be said, however, that perhaps the majority of patients swallow very well in the inclined position. Again, there is one danger that should be referred to in this connection; that is the danger of expulsion of the tube while in this position. Not long ago I had a little patient two years old, who was doing remarkably well. On the second day, while he was being fed in the inclined position, there was a sudden coughing spell, and soon after the people noticed that the child was breathing hard. They postponed sending for me for some little time, and when they did finally send, it was too late; the child was dead before I arrived. Upon examining the child, in the endeavor to remove the tube, I found it was not in the larynx, and upon searching it was found in the post nasal space. The tube had slipped down while the child was in the inclined position. This accident will not frequently occur, but it is a danger we must bear in mind. I think my success has been dependent, in no small degree, upon the use of small tubes. I invariably use a smaller tube than is appropriate for the age of the child. One of the greatest dangers is from the detachment of membrane below the tube, and I believe the fault with many who have no success with intubation is that they use too large a tube, one that fits too tightly. When detachment of membrane occurs the tube is not expelled, and suffocation quickly follows. Frequently I am called to patients to replace a tube that has been ejected, and I would much prefer to replace the tube several times than to take the risk of sudden suffocation.

Dr. W. E. Casselberry: I feel that in this connection still a word is necessary, although it has already been mentioned by Dr. Ingals, in reference to the crowding down of the membrane in the performance of intubation. This, in my experience, has been a more frequent accident than we would be led to suppose from statistics already given. Thrice it has happened to me; twice I have been able to perform immediate tracheotomy supervening the intubation, to relieve the condition, and once saw death rapidly ensue, more rapidly than it othewise would have done, when tracheotomy was refused by the friends. In a certain orphan asylum in this city, up to three years ago, tracheotomies had been performed, and death resulted in every instance. The management of that asylum thereupon resolved that no more operating in these cases would be permitted, and, as they expressed it, the patients would be allowed to die in peace. With the advent of intubation and the recent recurrence of cases of diphtheria in the institution, I was requested to perform this operation. The child was eight years of age; it was in all respects, to begin with, what would be regarded as a favorable case for tracheotomy. It was in the last stages of dyspnœa from laryngeal diphtheria. The introduction of the tube, under these circumstances, crowded the membrane down, and death was very rapidly imminent, so that hasty action was necessarily taken. The authorities of the institution had to be consulted before tracheotomy could be done; they finally agreed, after consultation with the mother of the child, that tracheotomy would be permitted. By that time the child had ceased respiration completely—was dead, although not more than ten minutes had elapsed from the performance of intubation. The child was hastily placed upon the table, and a tracheotomy tube inserted. The child was resuscitated, and finally recovered. I mention this case simply to urge the necessity, on the part of the physician when performing intubation, to have it thoroughly understood that tracheotomy may be immediately necessary to supplement the operation of intubation, and to have the instruments and table all ready to perform instantaneous tracheotomy in case the membrane is crowded down ahead of the tube.

Dr. Frank Billings: I would like to ask a question of Dr. Ingals or Dr. Casselberry. When Dr. Casselberry made known his method of feeding, he only described one form of inclining the patient—the head backward on an inclined plane, and like Dr. Waxham, I found some difficulty in feeding two recent patients in that way; but by turning them over on the abdomen with the head on an inclined plane, they took the food just as well. I would like to know if that method has been tried.

Dr. W. E. Casselberry: I would simply mention that I have tried both methods.

The one originally described was on the back. Following my publication and description of this method of feeding in the inclined plane position in cases of intubation, I received a letter from Dr. John Bartlett, who suggested feeding upon the abdomen in the inclined plane position. I had already, at that time, tried this method in one of the patients I was feeding on the back, but it did not seem to me to work as well as the dorsal position. Since then I have tried it and it seems to work equally well with the dorsal position. I think it is a matter of suitability to the individual case, whether the child should be inclined on the abdomen or on the back.

Dr. W. F. Coleman: I would like to ask a question on the pathology of post-diphtheritic paralysis about the eye, which has interested and puzzled me much. The paralysis of the external ocular muscles is said not to exist after diphtheria, but in my own observation it does exist. It is rare to have paralysis of accommodation without dilatation of the pupil, except in diphtheria. On the other hand, paralysis of the sphincter of the iris is commonly accompanied by paralysis of accommodation. Why we should have paralysis of the accommodation without paralysis of the sphincter is to me a query, and one I am not able to give an opinion upon. It would seem a very singular thing that diphtheria should select the ciliary nerves, which come from a very small ganglion to the ciliary muscle, and not attack the branches to the iris. As to the time: It appears that paralysis occurs usually within three to six weeks after the disease. And it occurs in very mild cases of diphtheria. As to the age, my observation is that a good many cases occur in young persons.

Dr. D. R. Brower: I do not know that I have anything to say in reply to the inquiry of Dr. Coleman, except to reiterate the statement that the pathology of post-diphtheritic paralysis is yet uncertain. We cannot explain these cases by any of the ordinary pathological conditions that give rise to paralysis. In the case the Doctor has mentioned, of accommodation paralysis, there must be something more than the neuritis that is considered the basis of most cases. It must be there is something developed by these micro-organisms, which plays havoc with the nervous system.

Dr. E. J. Doering: I would like to ask Dr. Ingals to compare the statistics of tracheotomy and intubation. Is it really fair to compare the two? Can the Doctor give us the statistics of twenty suffering from diphtheria, in which tracheotomy has been performed, and twenty of intubation? Such statistics would be more valuable for comparison than merely so many of intubation and so many of tracheotomy.

Dr. John W. Niles: I understand Dr. Ingals to say that tracheotomy in adults, after diphtheria, was uniformly fatal. I simply want to report that Dr. C. T. Parkes performed a tracheotomy on a friend of mine who recovered.

Dr. E. F. Ingals: With reference to Dr. Doering's question, I may say there is no fair comparison of intubation and tracheotomy, and there is no way to make a fair comparison until statistics from the new operation are much larger. We cannot know about the relation of cases in either instance, but we may make as fair comparison by taking the whole number of cases as in any other way. Some surgeons, who have had great success in tracheotomy, have advised this operation as soon as they noticed difficulty in breathing; some who have had success in intubation have done the same. Either operation performed early will be much more successful than if performed late. Either operation performed early will get the credit of saving patients who would have lived without it. There is no question in my mind but that the operation should be done early, and if tracheotomy is done as early as intubation, I presume the results will be much the same.

Dr. W. G. Dyas: When I first came to this city in 1859, diphtheria was very prevalent; twenty-one children died in immediate succession. These children were all under the mercurial treatment, and then for the first time, did I use the iron treatment. I use very largely the perchloride of iron. I gave large doses of iron internally, and large doses of wine as stimulants. There is no disease in which stimulants are so well borne as in diphtheria. The result was a good deal of success after a great deal of mortality from the disease. Some men were opposed to the use of tonic treatment, and would insist on going on with calomel in large doses. I think it was in 1859 or 1860 that I was called to Lyons, in consultation with Dr. Fox. On the way there I saw a child lying dead. It had recovered to a certain extent from diphtheria, was dressed and walking about, and every one supposed it had recovered from the disease; when suddenly it was seized with paralysis of the heart. In that case paralysis set in sooner than it generally does in this disease. I saw Dr. Fox, and to his credit be it said, he had no thought of anything but saving his patients. I proposed the iron treatment and stimulant and he said, "Do what you please, I am satisfied to see my patients recover." They were two German children, and are now young ladies, living in this city not far from my house. In their cases we used the perchloride of iron with equal parts of glycerine, applied internally to the fauces, and at the same time used muriate tincture of iron in ten minim drops every three hours. Wine was given in large quantities, and I was surprised to see children so young able to bear such large quantities of wine. I have treated as much diphtheria as most men in this city, and have had a great deal of success. I have scarcely changed my practice. One case I attended with a friend of mine was unsuccessful. In that case we did all that could be done from the beginning; injections of lime-water were made along the nostrils; lime-juice gargles were used, and large quantities of stimulants were given, but we had a very unruly child to deal with and it died; not from the treatment nor the disease, but from obstinacy. He died from paralysis of the heart. That was the only case that has been unsuccessful with me that I attended from the commencement. We now use carbolic acid vapor in the room and lime-water injections along the nostrils, and I have very few cases that terminate in affections of the larynx and trachea. I have been most fortunate in this respect. I cannot say one word about intubation or tracheotomy, for my cases have not gone so far. I can help them by decisive action in the beginning, and thus a majority of the cases will recover.

Dr. Ephraim Ingals: I have found nothing better in the treatment of diphtheria than to adhere to the old simple methods, and in therapeutics I still place my chief reliance on the tincture of chloride of iron, quinine, and chlorate of potassium. If the sick will hold the compressed tablets of the chlorate in the mouth and allow them to dissolve slowly—as they will—the remedy will be more efficacious than when prescribed in solution. Twenty to thirty grains should be administered daily in this way. Beyond what influence the agent may exert through the blood, this method gives it a long, continued application to the throat.

When patients are old enough to do it, they are benefited by frequent cleansing of the throat with gargles of warm water. I would isolate the sick when practicable. The importance of abundant and pure air, an even, and not too low temperature, good nutrition and caution during convalescence, is inculcated by all alike.

Dr. G. F. Lydston: There is one point in connection with catarrhal diphtheria that I think was not brought out as strongly as it should have been; that is, that some of the apparently mild cases are apt to be neglected, their importance underrated, and the patient allowed to go about too soon, as a consequence of which fatal, cardiac paralysis or other disagreeable consequences may ensue. A case illustrating this came under my observation in a prominent young business man in this city, who had a very slight attack of catarrhal diphtheria; in fact it was pronounced by one gentleman who saw him to be ordinary follicular pharyngitis. He was confined to the house about three days and then dismissed under the supposition that he was entirely well. He had previously been a perfectly healthy man, and, as was his custom prior to his illness, he took a cold bath before going down to business, and was found in the bath-room half an hour later dead, probably from paralysis of the heart. I think that case illustrates the care that should be taken in differentiating catarrhal diphtheria from simple sore throat. All cases that can not be accurately diagnosed, as well as cases that can be traced directly to infection, should be carefully watched.

Dr. Brower, in mentioning the various nervous sequelæ of diphtheria, failed to mention a peculiar symptom I have seen in two cases, viz.: paralysis of the bowels, although this may have been embraced in his remarks upon the effects of diphtheria on the spinal cord. I have seen two cases in young children. In one case in the third week, in the other the sixth week, I was called suddenly to see the little patients. I found the abdomen in each instance tremendously swollen. I have never seen cases of tympanites as marked as were these. The condition was associated with most obstinate constipation, and in each case with paraplegia. I ventured the opinion that the abdominal distention would disappear, as the other nervous symptoms improved, and my prediction proved correct—the condition disappearing in four or five weeks.

Dr. E. F. Ingals: I want to say one word with reference to instructing the community how to prevent diphtheria. I believe diphtheria is contagious, although there are many cases in which it does not seem to be so. I firmly believe that a great majority, perhaps more than half the cases of diphtheria, are caused by negligence of parents or friends about keeping the house properly warmed. A majority of the cases of diphtheria occur in mild weather, when it is a little too cold to be without fire, and a little too warm with it. As a result, many houses will be found at about 65°, and we will find the little ones running about, in and out of doors, dressed in their summer clothing. They necessarily take cold; they contract sore throat, and, with or without the bacillus as a cause, diphtheria is developed, whereas, if they were properly clothed, and the house was kept properly warmed, they would not have the disease.

I think the local treatment is not always as important as is thought by some physicians. It is well enough if it can be done without getting into a quarrel with the child. I recollect very well what Dr. Dyas told me, a few years ago, of his experience with the local treatment of diphtheria. The case was something like this: The child had diphtheria; the doctor believed in local treatment, and the father said we will apply it. The child died in the struggle, and I understood the Doctor that he has since given up local treatment unless the child accepts it willingly.

I believe that the tincture of the chloride of iron is one of our best internal remedies, and it may be given so frequently that a good local application is made every half hour, or less frequently, without resort to swabs, brushes, or sprays.

In regard to fatal cases, in which both intubation and tracheotomy have been performed, I am asked whether the result is charged to tracheotomy or intubation? I have no doubt they are charged to intubation by the tracheotomists, and to tracheotomy by the intubationists. Prof. Tirsch, of Liepzig, has performed intubation thirty-two times with only three recoveries. In fourteen of these cases he subsequently did tracheotomy, but the children died, and therefore, we are told, he abandoned intubation (because the children died in spite of tracheotomy). Would it not have been just as reasonable to abandon tracheotomy?

Dr. A. H. Foster: I invariably use chlorate of potash with the tincture of iron. In cases of the croupous form only, am I accustomed to give mercury; in the other forms of diphtheria I try to saturate the patient with tincture of iron and chlorate of potash solution.

